# Complete mitogenome of *Saccharina* cultivation variety ‘Ailunwan’(*Saccharina japonica* × *latissima*)

**DOI:** 10.1080/23802359.2016.1176878

**Published:** 2016-06-20

**Authors:** Jing Zhang, Tao Liu, Lei Zhang, Jing Liang

**Affiliations:** a Qilu University of Technology, Jinan, People’s Republic of China;; b College of Marine Life Sciences, Ocean University of China, Qingdao, People’s Republic of China

**Keywords:** Ailunwan, genetic differentiation, mitogenome, phylogenetic relationship

## Abstract

Complete mitogenome of *Saccharina* variety ‘Ailunwan’ (*Saccharina japonica* ×* latissima*) was obtained in this work. Circular-mapping mitogenome was 37,657 bp with overall A + T content of 64.71%, encoding 3 rRNAs (23S, 16S and 5S), 25 tRNAs, 35 protein-coding genes and 3 open reading frames (ORFs). Gene content and genome organization of ‘Ailunwan’ mitogenome were identical to those *Saccharina* species. From the total alignment of ‘Ailunwan’ and another reported variety ‘Rongfu’ (*S. japonica* ×* latissima*), 33 nucleotide substitutions were detected, and one intergenic region of 19 nucleotides insertion/deletion was found which located between *rps*3 and *rps*9. The phylogenetic analysis based on mitogenomes showed that ‘Ailunwan’ had a closer evolutionary relationship with *Saccharina* than *Laminaria* and validated the genus *Saccharina* and *Laminaria* were polyphyletic.

*Saccharina* (Laminariales, Phaeophyceae) is an important economic seaweed with respect to both its economic importance and global distribution (Kain 1979). ‘Ailunwan’ is one of the most important commercially *Saccharina* varieties in China. Here, we obtained the complete mitogenome of ‘Ailunwan’ (specimen number: 201004476, collected from Li dao Bay, Shandong, China and stored at −80 °C in the Culture Collection of Seaweed at Ocean University of China) via primer walking and long PCR techniques and gave the comparison with that of ‘Rongfu’, as part of our work to understand genetic characteristics of different varieties at genomic level.

The complete mitogenome of ‘Ailunwan’ was characterized as a circular molecule of 37,657 bp (GenBank accession number KU556731). The nucleotide composition was as follows: A = 10,700 (28.41%), C = 5541 (14.71%), G = 7749 (20.58%) and T = 136,667 (36.29%). The mitogenome had an overall A + T content of 64.71%. Cumulative GC-skew (0.1661) and AT-skew (–0.1218) analysis of mitogenome reflected a slight bias towards G and T in nucleotide composition on H-strand. The total intergenic regions were 2445 bp, accounting for 6.49% of the whole mitogenome.

The mitogenome encoded 66 genes, including 3 rRNAs, 25 tRNAs, 35protein-coding genes and 3 open reading frames (ORFs). The gene arrangement and component were identical within *Saccharina* mitogenomes (Yotsukura et al. 2010; Zhang et al. 2011; 2013), showing highly conservative evolution. Excepting *rpl*2, *rpl*16, *rps*3, *rps*19, *tat*c and ORF130, 60 genes were encoded on H-strand. All protein-coding genes started with ATG codon. Approximately 68.42% of protein-coding genes terminated with TAA codon, higher than that for TAG (21.05%) and TGA (10.53%). There were 13 pairs of genes with overlapping by 1–16 bp, making full use of nucleotide and genetic information. One obvious gene cluster (*rps*8-*rpl*6-*rps*2-*rps*4) was conserved in *Saccharina* mitogenomes.

Total mitogenome sequences of ‘Ailunwan’ and ‘Rongfu’ were aligned to find useful DNA barcoding. 33 nucleotide substitutions and 19 nucleotides intergenic region located between *rps*3 and *rps*9 in ‘Ailunwan’ while lacked from ‘Rongfu’ were found. Bayesian analysis based on combined 35 protein-encoding genes shared by Laminariaceae species exhibited the species were divided into two clades: *Saccharina* and *Laminaria* (Figure 1). Phylogenetic analyses showed that ‘Ailunwan’ firstly groups with *Saccharina japonica*. It indicated that ‘Ailunwan’ had a closer evolutionary relationship with *Saccharina* than *Laminaria*, supporting current taxonomic systems (Yoon et al. 2001; Lane et al. 2006).

**Figure 1.**
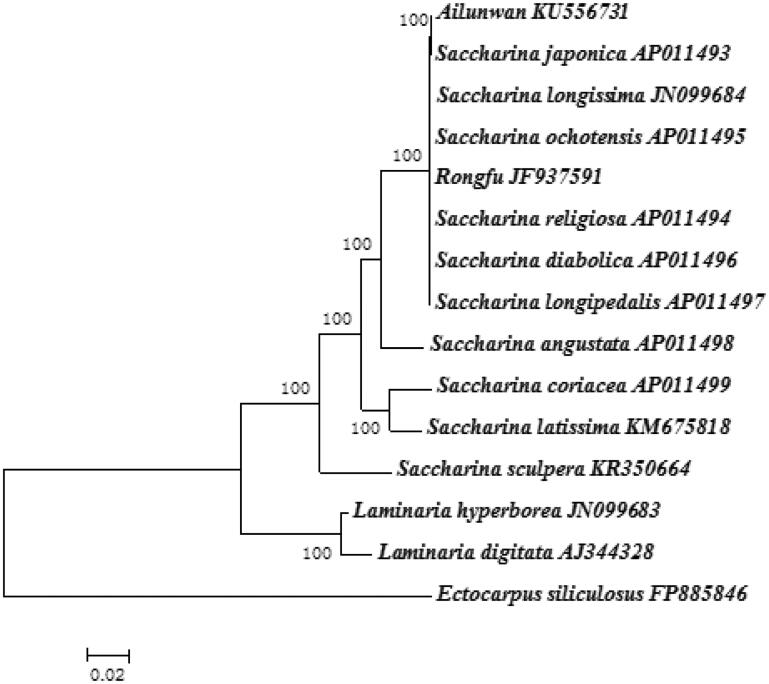
Phylogenetic trees derived from Bayesian analysis constructed based on concatenated nucleotide sequences of 35 mtDNA protein-encoding genes.
